# DNMT aberration-incurred GPX4 suppression prompts osteoblast ferroptosis and osteoporosis

**DOI:** 10.1038/s41413-024-00365-1

**Published:** 2024-12-02

**Authors:** Binjia Ruan, Jian Dong, Fanhao Wei, Zhiqiang Huang, Bin Yang, Lijun Zhang, Chuling Li, Hui Dong, Wangsen Cao, Hongwei Wang, Yongxiang Wang

**Affiliations:** 1 Department of Orthopedics, Northern Jiangsu People’s Hospital, Clinical Teaching Hospital of Medical School, Nanjing University, 98 West Nantong Road, Yangzhou, 225001 China; 2 Department of Thoracic Surgery, Nanjing Drum Tower Hospital, The Affiliated Hospital of Medical School, Nanjing University, Nanjing, Jiangsu 210008 China; 3 Nanjing University Medical School, Jiangsu Key Laboratory of Molecular Medicine & State Key Laboratory of Analytical Chemistry for Life Science, 22 Hankou Road, Nanjing, 210093 China; 4 Department of Respiratory Medicine, Jinling Hospital, Affiliated Hospital of Nanjing University Medical School, 305 East Zhongshan Road, Nanjing, 210002, China

**Keywords:** Bone, Osteoporosis, Pathogenesis

## Abstract

Osteoporosis (OP) is a common and fracture-prone skeletal disease characterized by deteriorated trabecular microstructure and pathologically involving various forms of regulated bone cell death. However, the exact role, cellular nature and regulatory mechanisms of ferroptosis in OP are not fully understood. Here, we reported that OP femurs from ovariectomized (Ovx) mice exhibited pronounced iron deposition, ferroptosis, and transcriptional suppression of a key anti-ferroptotic factor GPX4 (glutathione peroxidase 4). GPX4 suppression was accompanied by hypermethylation of the Gpx4 promoter and an increase in DNA methyltransferases DNMT1/3a/3b and was transcriptionally promoted by repressive KLF5 and the transcriptional corepressors NCoR and SnoN. Conversely, DNMT inhibition with SGI-1027 reversed promoter hypermethylation, GPX4 suppression and ferroptotic osteoporosis. In cultured primary bone cells, ferric ammonium citrate (FAC) mimicking iron loading similarly induced GPX4 suppression and ferroptosis in osteoblasts but not in osteoclasts, which were rescued by siRNA-mediated individual knockdown of DNMT 1/3a/3b. Intriguingly, SGI-1027 alleviated the ferroptotic changes caused by FAC, but not by a GPX4 inactivator RSL3. More importantly, we generated a strain of osteoblast-specific *Gpx4* haplo-deficient mice *Gpx4*^Ob+/−^ that developed spontaneous and more severe ferroptotic OP alterations after Ovx operation, and showed that GPX4 inactivation by RSL3 or semi-knockout in osteoblasts largely abolished the anti-ferroptotic and osteoprotective effects of SGI-1027. Taken together, our data suggest that GPX4 epigenetic suppression caused by DNMT aberration and the resulting osteoblastic ferroptosis contribute significantly to OP pathogenesis, and that the strategies preserving GPX4 by DNMT intervention are potentially effective to treat OP and related bone disorders.

## Introduction

Osteoporosis (OP) is a common and silent skeletal disease with a high risk of fragility fractures that affects approximately 18.3% of the globe populations, especially in postmenopausal women (11.6% of men and 23.1% of women).^1,2^ OP pathogenesis is characterized by reduced bone mass and deteriorated trabecular microstructure attributed directly to an imbalance of osteoclastogenesis and osteoblastogenesis.^3^ Under normal condition, bone undergoes constant turnover mainly controlled by osteoblasts and osteoclasts of opposite functions. Osteoclasts secret hydrochloric acid and proteolytic enzymes that dissolve organic collagen, inorganic calcium and phosphoruse, resulting in bone resorption.^4,5^ In contrast, osteoblasts produce various growth factors, hormones and collagens to ensure proper bone biosynthesis.^6,7^ OP occurs as a result of increased osteoclastogenesis or decreased osteoblastogenesis, or both.^8,9^ Although past researches have established that various forms of regulated cell death, such as apoptosis,^10^ autophagic cell death,^11^ pyroptosis,^12^ necroptosis.^13^ and ferroptosis^14,15^ contribute to OP pathogenesis, the precise role, cellullar nature and regulatory mechanisms of ferroptosis are only partially understood.

Ferroptosis is a unique form of regulated cell death caused by iron-dependent accumulation of lipid peroxides and is actively involved in various pathological conditions, including cancer, neurodegenerative diseases, cardiovascular and various bone diseases.^16–18^ Ferroptosis is not prevented by inhibitors of necroptosis, pyroptosis or apoptosis, but is inhibited by iron chelators and small lipophilic antioxidants such as ferrostatin^19^ and liproxstatin^20^ and directly regulated by endogenous glutathione GSH/GPX4 (glutathione peroxidase 4), the central anti-ferroptosis signaling pathway.^21^ GSH tripeptide consisting of glutamic acid, cysteine and glycine acts as a free radical scavenger and a cofactor of GPX4. Insufficient GSH generation reduces the synthesis of GPX4, which is able to convert the deleterious phospholipid hydroperoxides to the corresponding benign phospholipid alcohols, thus blocking ferroptosis.^22^ GPX4 suppression due to its reduced synthesis, enzymatic inactivation or protein degradation is a hallmark of ferroptosis.^23^ However, GPX4 transcriptional regulation through epigenetic or non-epigenetic regulations could also affect its abundance. The GPX4 promoter contains a dense CpG island, a structural feature of DNA methylation modification. GPX4 suppression due to DNA methylation affects its transcription under various ferroptotic conditions,^24,25^ suggesting that GPX4 expression in OP is likely subject to epigenetic DNA methylation controls, which represents a fundamental new mechanism of OP pathogenesis.

DNA methylation on cytosine of CpG dinucleotide (5-methylcytosine, 5mC) is a core epigenetic mechanism that potentially regulates the transcription of more than 60% of genes.^26,27^ DNA methylation is catalyzed by maintenance DNA methyltransferase DNMT1 and de novo DNMT3a and DNMT3b, while the demethylation is processed by three Ten-Eleven translocation enzymes TET1, TET2 and TET3.^28^ DNA methylation can occur at any CpG site along the genome, however its preferential modification of CpG islands in gene promoters/enhancers attracts DNA methylation readers, transcriptional repressors/cofactors and histone deacetylases to form a transcriptional repressive complex, resulting in silencing of the downstream gene transcription.^28^ DNA methylation-mediated suppressions of tumor suppressors, anti-aging proteins and cellular protective factors are common epigenetic features of tumorgenesis, aging and various degenerative and chronic diseases. Recent DNA methylation profiling investigations of OP bone tissues from animal models and OP patients detected a large number of genomic loci/genes that are modified by DNA methylation.^29–31^ More pertinently, DNA methylation-induced suppression of ferroptosis-associated genes *GPX4* and *CDH1* increases the ferrotosis sensitivity,^24,32^ strongly suggesting that epigenetic *GPX4* suppression due to DNA methylation aberration might mechanistically affect OP.

In this study, we aimed to investigate the role, cellular nature and regulatory network of ferroptosis in a mouse OP model of ovariectomy (Ovx). We discovered that GPX4 suppression and ferroptotic alterations in both Ovx mouse femurs and ferroptotic osteoblasts occurred substantially at mRNA levels that correlated with aberrant elevations of bioactive DNMT1/3a/3b. We then used both pharmacological and genetic approaches to determine the critical role of GPX4 suppression and ferroptosis in OP. Our study provides molecular insights into the epigenetic mechanisms of ferroptosis in OP pathogenesis with clinical prophylactic and therapeutic implications.

## Results

### Mouse osteoporotic femurs induced by ovariectomy display marked ferroptosis and GPX4 suppression

**Fig. 1:**
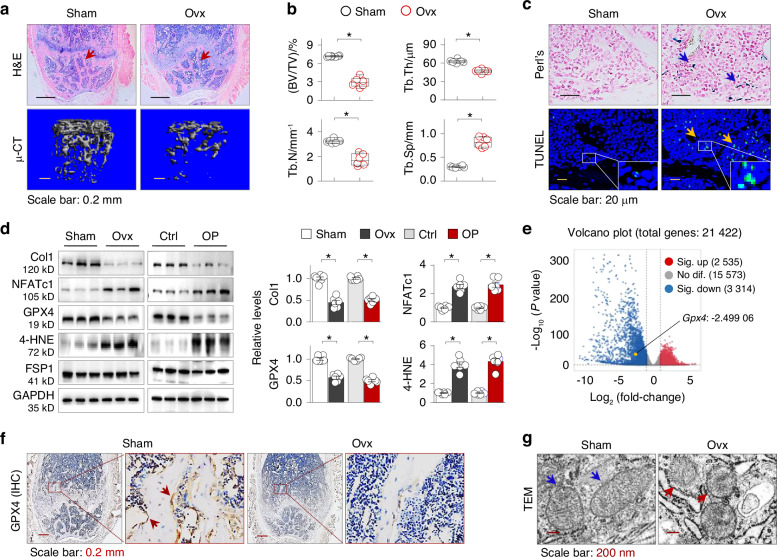
Mouse osteoporotic femurs induced by ovariectomy display marked ferroptosis and GPX4 suppression. **a** Representative microphotographs of H&E-stained distal femur sections (upper panel, arrows indicated trabeculae) and micro-CT-scanned (μ-CT) and 3-D reconstructed distal femurs from sham or Ovx mice (lower panel). Female C57BL/6 J mice of 12 weeks old were subjected to sham or ovariectomy (Ovx) surgery for 6 weeks (*n* = 6). **b** Quantitative analysis of μ-CT data in (**a**) for bone volume to tissue volume ratio (BV/TV), trabecular number (Tb.N), trabecular thickness (Tb.Th) and trabecular separation (Tb.Sp), presented as box-and-whisker plots. *P < 0.05, *n* = 6, Student’s *t* test. **c** Representative distal femur sections stained by Perls’ method (upper panel, arrows indicate iron deposition) and by TUNEL staining (lower panel, arrows indicate positively-stained cells). **d** Western blotting. Femur homogenates from Sham and Ovx mice, control (Ctrl) and OP patients were assayed for type 1 collagen (Col 1), NFATc1, GPX4, 4-HNE and FSP1. GAPDH served as control. Three randomly-selected samples from 6 in each group were shown. Quantification of (**d**) on the right side of the blots was presented as mean ± SEM. *P < 0.05, Student’s *t* test. **e** Volcano plot of gene expression profile from database CRA007214 (total 21 422 genes, *n* = 3 mice for Sham and Ovx group). The number and position of genes statistic-significantly increased (2 535, red), no difference (15 573, gray) or decreased (3 314, blue) including *Gpx4* (Log_2_ fold change, FC = –2.499 06, yellow point) were marked. **f** Representative distal femur sections of Sham and Ovx mice stained for GPX4 by immunohistochemical (IHC) staining. The images on the right side of each panel were enlarged frames of the left panel. Positively-stained cells in brown were indicated by arrows. **g** Representative TEM micrographs of the femurs of Sham and Ovx mouse. Normal and ferroptotic mitochondria were indicated by blue and red arrows, respectively

To gain insight into the possible epigenetic GPX4 suppression and ferroptosis in OP, we employed a well-established mouse OP model of ovariectomy (Ovx).^33^ As expected, mice receiving Ovx surgery for 6 weeks exhibited thinned trabeculae with disrupted continuity and enlarged areolae in distal femurs as evidenced by H&E staining (Fig. 1a). Microcomputed tomography (μCT) scans revealed that OP femurs had reduced trabecular bone volume relative to tissue volume ratio(BV/TV), decreased trabecular bone number (Tb.N), reduced trabecular thickness (Tb,Th) and increased trabecular bone separation (Tb.Sp) (Fig. 1a, b). Perls’ Prussian blue staining, which detects hemosiderin-associated Fe^3+^, showed increased iron deposition (Fig. 1c), and TUNEL staining, a sensitive assay to catch both apoptotic and ferroptotic cells,^20^ detected an increase in TUNEL- positive cells (Fig. 1c).

We further performed western blotting assays and detected a decrease in the osteoblast marker type 1 collagen and an increase in the osteoclast marker NFATc1 (Nuclear Factor Of Activated T Cells 1), a typical sign of osteoporosis. Notably, GPX4, a key anti-ferroptosis enzyme, was suppressed, accompanied by an increase in the lipid peroxidation marker 4-hydroxynonenal (4-HNE). In contrast, the expression of another anti-ferroptosis protein FSP-1 (ferroptosis suppressor protein 1), which traps lipid peroxyl radicals independent of GSH/GPX4 signaling,^34^ was unaffected (Fig. 1d). We also analyzed RNA-seq data from a CNCB (China National Center for Bioinformation) database^35^ and found 2 535 upregualted and 3 314 down-regulated genes, and confirmed the downregulation of Gpx4 in tibia of Ovx mice (Log_2_ fold change = -2.499 06, Fig. 1e). Further, immunohistochemical (IHC) staining of femur sections revealed that GPX4 was broadly expressed in nearly all visible cells around the femoral trabeculae, where osteoblasts, bone lining cells and osteoclasts often reside, but the levels were drastically decreased in Ovx mice (Fig. 1f). Finally, transmission electron microscopy (TEM) confirmed that OP femurs of Ovx mice exhibited distinct characters of ferroptotic mitochondrial alterations, such as smaller mitochondria and diminished mitochondrial crista (Fig. 1g). These results indicate that OP pathogenesis is associated with significant GPX4 suppression and ferroptosis.

### GPX4 suppression is mainly due to aberrant DNMT elevations and GPX4 promoter hypermethylation

**Fig. 2:**
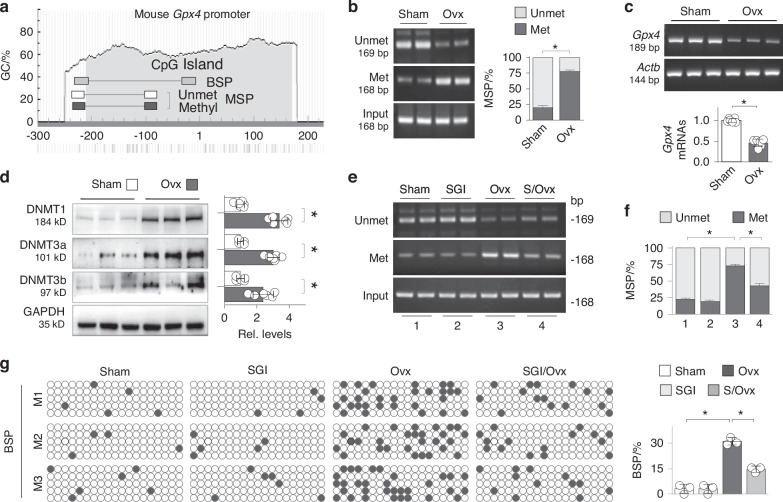
GPX4 suppression is mainly due to aberrant DNMT elevations and GPX4 promoter hypermethylation. **a** A schematic diagram of the mouse *Gpx4* promoter. The position of CpG island (gray area) and MSP/BSP primers (boxes) were depicted relative to the transcription starting site. **b** Representative agarose gel analyses of MSP products from femurs of Sham and Ovx mice (*n* = 6, 6 weeks). Two random samples from each group were shown. Quantification on the right was presented as mean ratio±SEM of methylated/unmethylated over total PCR products after adjusted with input control. *P < 0.05, Student’s *t* test. **c** RT-PCR and agarose gel analysis of *Gpx4* mRNA from femurs of Sham and Ovx mice (*n* = 6, 6 weeks). Beta-actin gene *Actb* served as internal control. Three random samples from each group were shown. Quantification below was presented as mean ± SEM. *P < 0.05, Student’s *t* test. **d** Western blotting of femur homogenates from Sham and Ovx mice for DNMT1, DNMT2 and DNMT3 with GAPDH serving as loading control. Three random samples from each group were shown (*n* = 6, 6 weeks). Quantification on the right side was presented as mean ± SEM. *P < 0.05, Student’s unpaired *t* test. **e** MSP. Femurs of Sham, SGI-1027 (SGI, 2.5 mg/kg daily), Ovx and SGI-1027-treated Ovx mice (S/Ovx) for 6 weeks (*n* = 6) were analyzed by MSP. Two representative samples from each group were shown. **f** Quantification of (**e**). Data were presented as mean ratio ± SEM of methylated/unmethylated over total PCR products after adjusted with input control. *P < 0.05, two-way ANOVA. **g** BSP analysis of the mouse *Gpx4* promoter. Three randomly-selected mice (M1, M2, and M3) from each group were subjected to BSP. Five cloned PCR products from each animal sample were sequenced. One box represented one mouse. Each box row represented one single sequenced clone and each dot represented one CpG site. Empty or dark dots indicated unmethylated or methylated CpGs, respectively. Quantification on the right side was presented as mean ratio ± SEM of methylated CpGs over total CpGs in the cloned fragments. ^*^ *P* < 0.05, two-way ANOVA

**Fig. 3:**
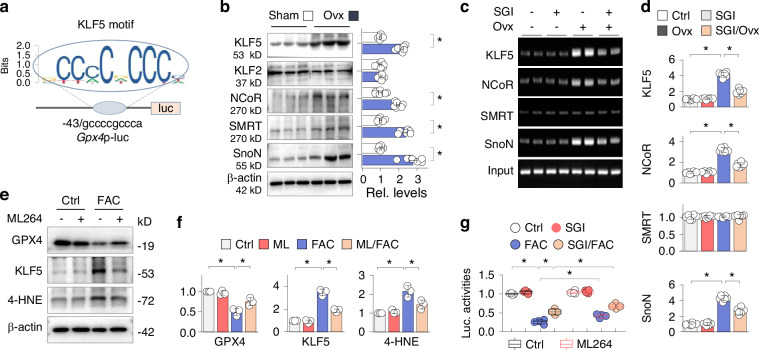
KLF5 is a potential co-regulator of Gpx4 transcriptional inhibition. **a** A schematic diagram of the *Gpx4* promoter/luciferase reporter *Gpx4*p-luc with KLF5 motif relative to the indicated transcriptional starting site. **b** Western blotting of sham and Ovx mouse femurs (6 weeks) for KLF5, KLF2, NCoR, SMRT and SnoN, with β-actin serving as internal control. Three random samples from each group (*n* = 6) were shown. Quantification on the right was presented as mean ± SEM. *P < 0.05, Student’s *t* test. **c** ChIP assay. The same femoral homogenates treated as above were first immunoprecipitated with antibodies to KLF5, NCoR, SMRT, or SnoN, separately, and then the precipitated DNA fragments were amplified by PCR with primers covering the KLF5 motif region. Non-immunoprecipitated DNA served as control (input). Two representative PCR products were shown on agarose gel. **d** Quantification of (**c**). Data were presented as mean ± SEM (*n* = 6). *P < 0.05, two-way ANOVA. **e** Western blotting. Primary osteoblasts were treated with FAC (100 μmol/L) in presence or absence of ML264 (10 μmol/L) for 48 h, and then cell lysates were assayed for GPX4, 4-HNE and KLF5 with β-actin serving as control. **f** Quantification of (**e**). Data were presented as mean ± SD of three repeated assays. *P < 0.05, two-way ANOVA. **g** Luciferase assay. The *Gpx4* promoter/luciferase reporter plasmid and a Rennila luciferase reporter plasmid were co-transfected into primary osteoblasts, treated with FAC (100 μmol/L) with or without SGI-1027 (10 μmol/L) in the presence or absence of ML264 (10 μmol/L) for 48 h, and then the luciferase activities of *Gpx4*p-luc were measured, normalized with Renilla’s and presented as box-and-whisker plots of four repeated assays, *P < 0.05, two-way ANOVA

To investigate the possible role of DNA methylation in GPX4 suppression, we analyzed the mouse and human *Gpx4*/*GPX4* promoters using MethPrimer (http://www.urogene.org/methprimer) and found that both promoters contained conserved CpG islands at −210/170 (mouse) and −253/47 (human) loci relative to the transcription starting site (Fig. 2a and Fig. S1a), both were hypermethylated in Ovx mice and OP patients compared to controls [(77.45 ± 4.32)% of Ovx vs (20.15 ± 2.74)% of Sham, and (72.53 ± 2.81)% of OP vs (21.89 ± 2.63)% of Normal, respectively, *P* < 0.05, Fig. 2b and Fig. S1d], suggesting that GPX4 is sensitive to DNA methylation modification under OP conditions. Further RT-PCR confirmed that *Gpx4*/*GPX4* mRNAs were substantially reduced in bone tissues of Ovx mice and OP patients (Fig. 2b and Fig. S1b). Since DNMTs positively regulate the promoter methylation and gene transcription suppression, we then assessed the expression of DNMTs by western blotting and found that all three bioactive DNA methyltransferases DNMT1, DNMT3a and DNMT3b were relatively low in control femurs, but significantly upregulated in Ovx mice and OP patients (Fig. 2c and Fig. S1c). However, administration of a DNMT inhibitor, SGI-1027, significantly decreased the methylation level of the *Gpx4* promoter [(43.07 ± 3.44)% of SGI/Ovx vs (73.03 ± 2.99)% of Ovx, *P* < 0.05, Fig. 3e] in Ovx mice. To confirm these results, we performed BSP, the gold standard of DNA methylation measurement that detects individual CpG sites. The results showed that *Gpx4* promoter methylation increased from (3.19 ± 1.57)% to (31.23 ± 1.26)% in Ovx mice, but SGI-1027 treatment lowered the level to (14.74 ± 1.05)%, *P* < 0.05 (Fig. 3f). These results indicate that GPX4 suppression is mainly caused by DNMT1/3a/3b aberrations and the resulting promoter hypermethylation.

### KLF5 is a potential co-regulator of Gpx4 transcriptional inhibition

To understand the regulatory mechanisms underlying DNMT-incurred *Gpx4* transcriptional suppression, we analyzed the mouse *Gpx4* promoter using AnimalTFBD (http://bioinfo.life.hust.edu.cn/AnimalTFDB#!), which predicts the potential binding sites for transcriptional and co-transcriptional regulators. The analysis revealed numerous potential binding motifs for various transcriptional factors, including Sp1, C/EBPβ, NF-1C, and transcriptional co-repressors known to participate in epigenetic gene transcriptional inhibition such as NCoR, SMRT and SnoN. Notably, a Kruppel- like factor 5 (KLF5) motif was identified near the transcription starting site with a high binding score (-43/gccccgccca, score 19.303, Fig. 3a). KLF5 plays critical roles in various cancers^36^ and can either positively or negatively regulate downstream gene transcription.^37,38^ However its role in ferroptosis or bone metabolisms remains largely unknown.

We found that the expressions of KLF5, but not KLF2 with similar binding specificity, as well as the transcriptional co-repressor NCoR, SMRT and SnoN, were upregulated in Ovx femurs (Fig. 3b). We then performed a chromatin immunoprecipitation (ChIP) assay and found that KLF5, together with NCoR and SnoN, but not KLF2 or SMRT, inducibly bound to the KLF5 motif region of the *Gpx4* promoter in femurs of Ovx mice. However, SGI-1027 treatment significantly reduced the bindings (Fig. 3c, d). Furthermore, primary osteoblasts treated with ferric ammonium citrate (FAC) known to mimic iron overloading and induce ferroptosis^39^ also showed KLF5 upregulation, GPX4 suppression and 4-HNE induction, but these abnormalities were significantly reduced by a KLF5-selective inhibitor, ML264 (Fig. 3e, f). In addition, we also constructed a *Gpx4* promoter/luciferase reporter plasmid *Gpx4*p-luc (Fig. 3a) and observed that FAC treatment reduced the *Gpx4*-luciferase activity of *Gpx4*p-luc, which was partially alleviated by SGI-1027 and further relieved by ML264 (Fig. 3g). Taken together, these results suggest that DNMT aberration*-*induced *Gpx4* transcription suppression is at least partially mediated by KLF5, likely involving the transcription corepressors NCoR and SnoN.

### DNMT inhibition by SGI-1027 derepresses GPX4 and protects Ovx mice from ferroptotic osteoporosis

**Fig. 4:**
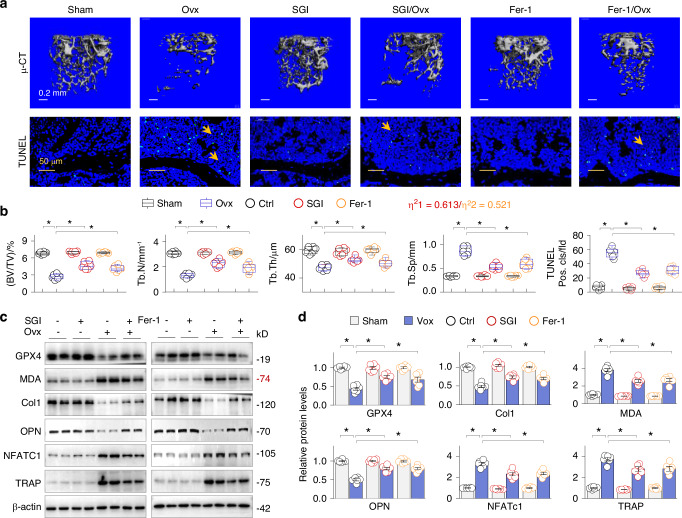
DNMT inhibition by SGI-1027 preserves GPX4 and protects Ovx mice from ferroptotic osteoporosis. The female C57BL/6 J mice were divided into Sham, SGI-1027 (SGI, 2.5 mg/kg daily) or ferropstatin-1 (Fer-1, 5 mg/kg daily), Ovx, SGI/Ovx or Fer-1/Ovx groups (*n* = 6, 6 weeks). **a** Representative photomicrographs of μ-CT-scanned (upper panel) or TUNEL-stained (lower panel, the positively-stained cells were indicated by arrows) mouse distal femurs/femoral sections. **b** Quantitative analysis of (**a**) for bone volume to tissue volume ratio (BV/TV), trabecular number (Tb. N), trabecular thickness (Tb. Th), trabecular separation (Tb. Sp) and TUNEL-positive cell counts. Data were presented as box-and-whisker plots. *P < 0.05, *n* = 6, two-way ANOVA. The effect size of the interaction between OP intensity (BV/TV ratio) and SGI-1027 (η^2^1) or Fer-1(η^2^2) intervention was indicated. **c** Western blotting of the femoral homogenates for GPX4, MDA, Col1, OPN, NFATc1 and TRAP. Two-samples from each group were shown. **d** Quantifications of (**c**). Data were presented as mean ± SEM. *P < 0.05, two-way ANOVA

To further investigate the functional relevance of GPX4 suppression and ferroptosis in OP pathogenesis, we treated control and Ovx mice with or without SGI-1027 (6 weeks, *n* = 6 per group). The results showed that SGI-1027 effectively corrected Ovx-induced osteoporotic alterations, including trabecular bone volume to tissue volume ratio (BV/TV), trabecular bone number (Tb.N), trabecular thickness (Tb,Th) and bone separation (Tb.Sp). Furthermore, TUNEL assays showed a reduced number of dead cells in the SGI-1027-treated group (Fig. 4a, b). Consistently, SGI-1027 significantly mitigated the adverse expressions of GPX4 and the lipid peroxidation marker MDA (malondialdehyde), as well as the osteoblast markers collagen 1 (Col1) and osteopontin (OPN) and the osteoclast markers NFATc1 and TRAP (Fig. 4c, d) in Ovx mice. Moreover, ferropstatin-1 (Fer-1), a lipophilic antioxidant that specifically inhibits ferroptosis by preventing lipid peroxidation without affecting other forms of regulated cell death,^19^ also achieved similar, albeit less effective, anti-ferroptotic and osteoprotective effects (Fig. 4a–d). We also calculated the effect sizes (η^2^) of the interactions between OP intensity (BV/TV ratio) and SGI-102 or Fer-1 interventions and found that SGI-1027 (η^2^1 = 0.613) compared to Fer-1 (η^2^2 = 0.521) had a stronger capacity (Fig. 4b, inset). These results suggest that DNMT aberration and the resulting GPX4 suppression and ferroptosis are central to OP pathologies.

### GPX4 suppression and ferroptosis occur mainly in osteoblasts

**Fig. 5:**
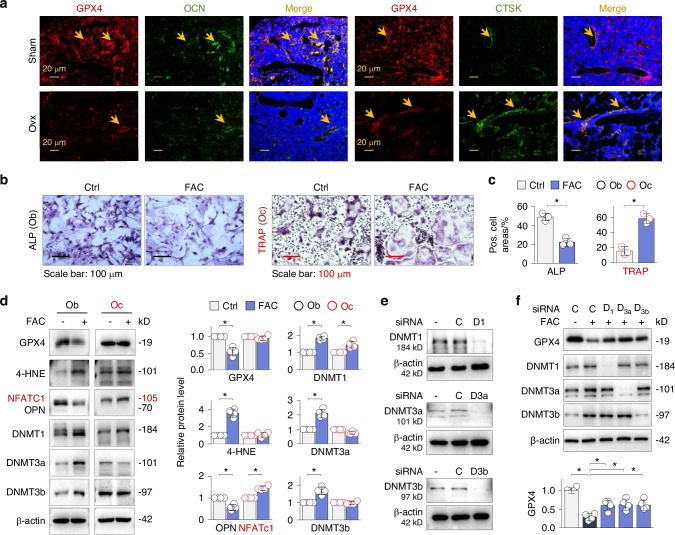
GPX4 suppression occurs mainly in osteoblasts and is mediated by all three DNMT isoforms. **a** Immunofluorescent double-staining. The distal femur sections of Sham or Ovx mice were double-stained for GPX4 (red) plus OCN or CTSK (green), respectively, counterstained with DAPI (blue) and then merged. The single or double-stained cells in yellow were indicated by arrows. **b** Representative images of osteoblasts stained for ALP (alkaline phosphatase, left panel) activity and osteoclasts stained for TRAP (tartrate-resistant acid phosphatase, right panel) activity. Quantification on the right was presented by mean percentages of positively-stained area of total area ± SD, *n* = 3, *P < 0.05, Student’s *t* test. **c** Western blotting. Primary osteoblasts (Ob) and osteoclasts (Oc) treated with or without FAC (100 μmol/L) for 48 h were examined for GPX4, 4-HNE, OPN (Ob), NFATc1 (Oc), DNMT1, DNMT3a and DNMT3b. β-actin served as controls. **d** Quantifications of (**c**). Data were presented as mean ± SD of 4 replicated experiments. *P < 0.05, Student’s *t* test. **e** Western blotting. Primary osteoblasts were transfected with siRNA-control(C), siRNA-DNMT1(D1), siRNA-DNMT3a (D3a) or siRNA-DNMT3b (D3b) for 24 h, and assayed for DNMT1, DNMT3a and DNMT3b, respectively. **f** Western blotting. The siRNA-transfected osteoblasts were treated with or without FAC (100 μmol/L), and then the cell lysates were assayed for GPX4 and DNMT1, DNMT3a and DNMT3b. Quantification of GPX4 below the blots was presented as mean ± SD of 4 replicated experiments. *P < 0.05, Student’s t test

To explore the cellular nature of GPX4 suppression and ferroptosis, we performed immunofluorescent double-staining of femur sections with GPX4 and either the osteoblast marker OCN (osteocalcin) or the osteoclast marker CTSK. The results showed that GPX4 was co-expressed with OCN-positive osteoblasts, and both signals decreased in Ovx femurs (Fig. 5a, left panel). In contrast, while CTSK-positive osteoclasts were barely detectable with discernable GPX4 coexpression in normal femurs, the staining increased in Ovx femurs with GPX4 expression remaining relatively high (Fig. 5a, right panel), suggesting that GPX4 suppression mainly occurs in osteoblasts.

We further isolated and cultured primary osteoblasts and osteoclasts (differentiated from bone marrow monocytes/macrophages induced by RANKL) and treated both cells with ferric ammonium citrate (FAC) that mimics iron overloading. FAC treatment decreased alkaline phosphatase activities (ALP) in osteobalsts but increased tartrate-resistant acid phosphatase (TRAP) activities in osteoclasts (Fig. 5b). Further, FAC induced similar GPX4 suppression, 4-HNE induction and OPN reduction accompanied by increases in all three DNMT isoforms in osteoblasts, but only elevated DNMT1 and NFATc1 in osteoclasts (Fig. 5c, d). To confirm the specific role of each DNMT isoform in inducing GPX4 suppression, we designed three siRNAs specifically targeting DNMT1, DNMT3a or DNMT3b (Fig. 5e) and found that knockdown of DNMT1, DNMT3a or DNMT3b each still significantly reversed FAC-induced GPX4 suppression (Fig. 5f), suggesting all three DNMT isoforms contribute to ferroptotic GPX4 suppression in osteoblasts.

### GPX4 inactivation by RSL3 blunts the anti-ferroptosis effects of DNMT inhibition

**Fig. 6:**
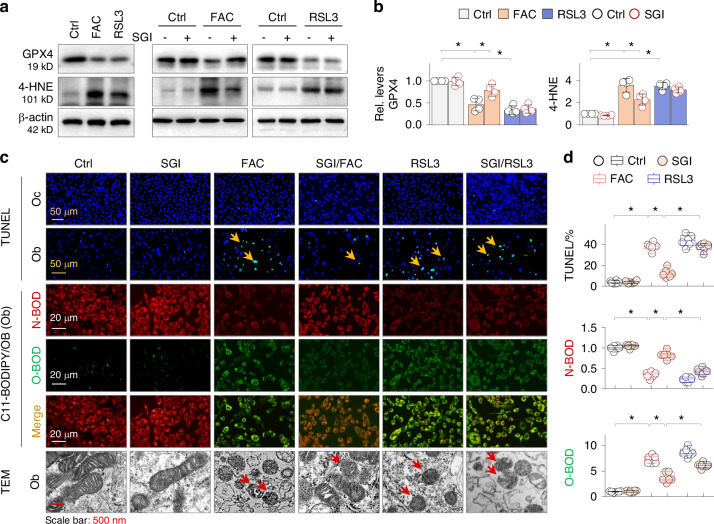
GPX4 inactivation by RSL3 blunts the anti-ferroptosis effects of DNMT inhibition in osteoblasts. **a** Western blotting. Primary osteoblasts treated with FAC (100 μmol/L) or RSL3 (0.3 μmol/L) with or without SGI-1027 (SGI, 10 μmol/L) for 48 h were assayed for GPX4 and 4-HNE. **b** Quantification of (**a**). Data were presented as mean ± SD. *P < 0.05, *n* = 4, two-way ANOVA. **c** Representative images of TUNEL staining, C11-BODIPY and TEM studies. Primary osteoblasts (Ob) and osteoclasts (Oc) treated as above were examined by TUNEL staining (upper panel, positive cells were indicated by arrows). The osteoblasts (Ob) were examined with C11-BODIPY (middle panel. N-BOD, non-oxidized-BODIPY: O-BOD, oxidized-BODIPY), and TEM (bottom panel, arrows indicated ferroptotic mitochondria). **d** Quantifications of (**c**). Data were presented as mean ratio ± SD (*n* = 6), *P < 0.05, two-way ANOVA

To ascertain the critical role of GPX4 preservation by SGI-1027 in mitigating osteoblastic ferroptosis, we treated osteoblasts with FAC or RSL3, a small molecule that induces ferroptosis by inactivating and degrading GPX4.^20^ Both FAC and RSL3 induced GPX4 suppression and 4-HNE induction (Fig. 6a). However, SGI-1027 significantly corrected these abnormalities induced by FAC, but not by RSL3 (Fig. 6a, b). To confirm osteoblast ferroptosis, we performed a TUNEL assay and found that FAC and RSL3 induced a significant increase in TUNEL-positive osteoblasts, but not osteoclasts. SGI-1027 treatment reduced the positive cell counts of osteoblasts induced by FAC, but had no effect on those induced by RSL3 (Fig. 6c, upper two panels, and 6d). We further assessed lipid peroxidation status of osteoblasts using C11-BODIPY, a fluorescent radio-probe that monitors lipid peroxidation in live cells.^40^ The results showed that resting osteoblasts displayed prominent non-oxidized BODIPY (N-BOD) and barely detectable oxidized BODIPY (O-BOD). FAC or RSL3 treatment caused an inversion of O-BODIPY/N-BODIPY distributions; however, SGI-1027 effectively corrected the inversion caused by FAC, but not by RSL3 (Fig. 6c, middle three panels). Additionally, transmission electron microscopy (TEM) revealed that FAC and RSL3 induced typical ferroptotic alterations in osteoblasts, such as smaller mitochondria and dismissed crista. However, SGI-1027 alleviated these alterations induced by FAC, but not by RSL3 (Fig. 6c, bottom panel). These results collectively suggest that under osteoporotic conditions, osteoblasts, but not osteoclasts, undergo ferroptosis that are regulated by DNMT1/3a/3b aberrations and GPX4 suppression.

### Osteoblast Gpx4 deficiency potentiates ferroptotic osteoporosis

**Fig. 7:**
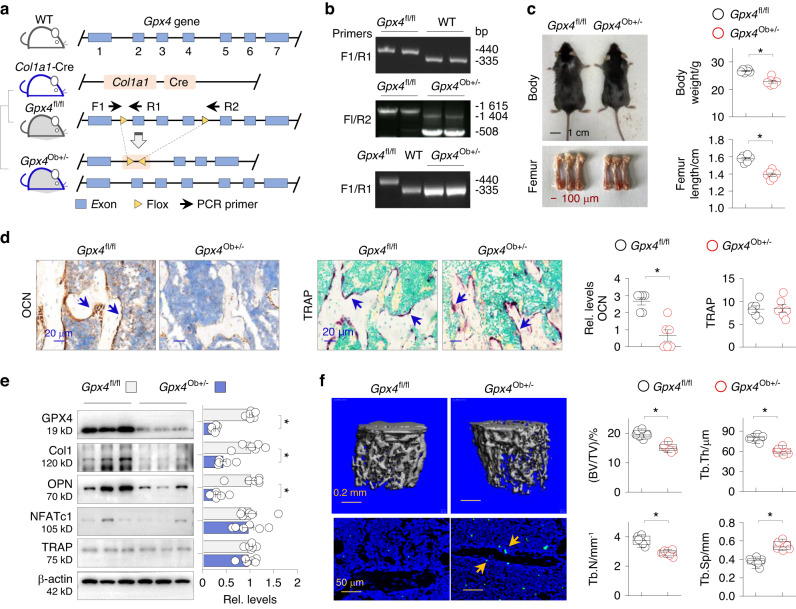
Osteoblastic *Gpx4* deficiency potentiates ferroptotic osteoporosis. **a** Generation of *Gpx4*^Ob*+/−*^ mice by crossing *Col1a1-Cre* with *Gpx4*^fl/fl^ mice. *Gpx4* gene in wild-type mice contains seven exons represented by boxes (1-7). The positions of the flox sites (triangles) in *Gpx4*^fl/fl^ mice and the genotyping PCR primers F1, R1, and R2 (arrows) were depicted. **b** Agarose gel representation of genotyping of WT, *Gpx4*^fl/fl^ and *Gpx4*^Ob+/–^ mice. **c** The appearance (upper panel) and femurs (lower panel) of *Gpx4*^fl/fl^ and *Gpx4*^*+/–*^ mice at 8 weeks. Quantification of mouse body weight and femur length on the right side (6 mice in each group) was presented as mean ± SEM. *P < 0.05, Student’s *t* test. **d** Representative photomicrographs of femur sections from *Gpx4*^fl/fl^ and *Gpx4*^Ob+/–^ mice stained for OCN (IHC) and TRAP activity. Arrows indicated the positively-stained osteobalsts (left panel) or osteoclasts (right panel). Quantification on the right side was presented as mean ± SEM. *P < 0.05, Student’s *t* test. **e** Western blotting of femoral homogenates from *Gpx4*^fl/fl^ and *Gpx4*^Ob*+/–*^ mice for GPX4, Col1, OPN, NFATc1 and TRAP. Quantification on the right side of each blot was presented as mean ± SEM. *P < 0.05, Student’s *t* test. **f** Representative photomicrographs of μ-CT-scanned (upper panel) and TUNEL-stained (lower panel) distal femurs/femoral sections of *Gpx4*^fl/fl^ and *Gpx4*^Ob*+/*−^ mice. Quantitative analysis of μ-CT 3D reconstruction data in (**e**) (right side) for bone volume to tissue volume ratio (BV/TV), trabecular number (Tb.N), trabecular thickness (Tb.Th) and trabecular separation (Tb.Sp). Data are presented as box-and-whisker plots. *P < 0.05, *n* = 6, Student’s *t* test

To directly test the role of osteoblast *Gpx4* suppression in ferroptotic osteoporosis, we generated a strain of osteoblast-specific *Gpx4* knockout mice by crossing *Gpx4*^fl/fl^ mice with *Col1a1*-Cre mice, supposedly resulting in the deletion of exons 2–4 of the *Gpx4* gene in osteoblasts (Fig. 7a). Previous study indicated that globe *Gpx4* gene knockout was embryonically lethal.^41^ We genotyped over 70 offsprings and found either wild-type or heterozygous *Gpx4*^Ob+/–^ mice, but no homozygous *Gpx4*^Ob–/–^ mice (Fig. 7b), indicating that complete *Gpx4* depletion in osteoblasts is also lethal. These *Gpx4*^Ob+/–^ mice at 8 weeks were smaller, had lighter body weights and shorter femurs (Fig. 7c). Staining of the osteoblast marker OCN (IHC) and osteoclast TRAP activity confirmed reduced OCN but unchanged TRAP intensities (Fig. 7d). Western blotting showed reduced femoral GPX4 and adverse expressions of the osteoblastic markers OPN and collagen1, but not the osteoclast markers NFATc1 and TRAP compared to *Gpx4*^fl/fl^ control (Fig. 7e). Additionally, these *Gpx4*^Ob+/–^ mice displayed altered osteoporotic parameters such as decreased bone volume/tissue ratio (BV/TV), trabecular number (Tb.N), trabecular thickness (Tb.Th), and increased trabecular separation (Tb.Sp) (Fig. 7f), and increased TUNEL-positive cell counts in distal femurs (Fig. 7f, left lower panel), supporting that GPX4 haplo-deficiency potentiates osteoblastic ferroptosis and OP pathologies.

### GPX4 preservation is essential for the anti-ferroptotic and anti-osteoporotic effects of SGI-1027

**Fig. 8:**
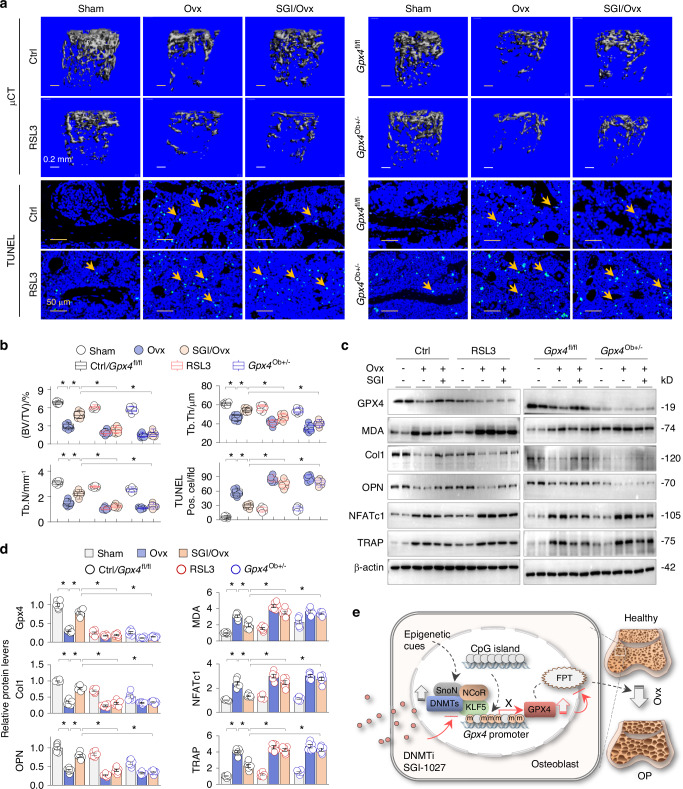
GPX4 presevation is essential for the anti-ferroptotic and anti-osteoporotic effects of SGI-1027. Control and RSL3-treated mice, as well as *Gpx4*^fl/fl^ and *Gpx4*^Ob+/–^ mice, were subjected to Sham, Ovx or SGI-1027/Ovx treatment for 6 weeks, respectively (*n* = 6). **a** Representative photomicrographs of the μ-CT-scanned (upper paenl) and TUNEL-stained (lower panel. Arrows indicated the positive cells) distal femurs/femoral sections. **b** Quantitative analysis of bone volume to tissue volume ratio (BV/TV), trabecular number (Tb.N), trabecular thickness (Tb.Th) and TUNEL staining in (**a**). Data were presented as box-and-whisker plots. *P < 0.05, two-way ANOVA followed by Tukey’s post hoc test. **c** Western blotting of mouse femoral homogenates in (**a**) for GPX4, MDA, Col1, OPN, NFATc1 and TRAP. Two random samples from each group were shown. **d** Quantification of (**c**). Data were presented as mean ± SEM. *P < 0.05, *n* = 6, Two-way ANOVA. **e** A schematic diagram of sequential DNMT1/3a/3b elevations, GPX4 promoter hypermethylation associated with KLF5, NCoR and SnoN, GPX4 transcriptional suppression and osteoblast ferroptosis (FPT) that promotes osteoporosis (OP) in Ovx mice (dashed lines). On the other hand, DNMT inhibition (DNMTi) with SGI-1027 blocks GPX4 suppression and ferroptotic osteoporosis (solid lines)

DNMT aberrations and SGI-1027 intervention likely impact the transcription of numerous genes. We hypothesized that if GPX4 preservation by SGI-1027 is critical for its anti-ferroptotic and anti-osteoporotic effects, then blocking GPX4 reactivation would negate these protective effects. To test this hypothesis, we compared the anti-osteoporotic effects of SGI-1017 between control and RSL3-treated mice, as well as between *Gpx4*^fl/fl^ and *Gpx4*^Ob+/−^ mice. Both RSL3-treated and *Gpx4*^Ob+/−^ mice exhibited adverse femoral osteoporotic changes, including trabecular bone volume to tissue volume ratio (BV/TV), trabecular bone number (Tb.N), trabecular thickness (Tb,Th) and increased TUNEL-positive cells under basal and Ovx conditions (Fig. 8a, b). SGI-1027 treatment significantly improved the bone microstructural parameters and TUNE-positive cell counts in control and *Gpx4*^fl/fl^ mice; however these protective effects were largely abrogated in RSL3-treated and *Gpx4*^Ob+/−^ mice (Fig. 8a, b). Similarly, SGI-1027 significantly reduced abnormal levels of GPX4, MDA, collagen I, OPN, NFATc1 and TRAP in control and *Gpx4*^fl/fl^ mice, but the beneficial effects were largely abolished in RSL3-treated and *Gpx4*^+/−^ mice (Fig. 8c, d). These results indicate that GPX4 preservation is essential for the anti-ferroptotic and osteoprotective effects of SGI-1027.

## Discussion

The study of epigenetic ferroptosis in OP represents a unique approach to better understand the OP pathogenesis and potentially leads to novel therapeutic strategies. In this study, we have discovered that (1) GPX4, a key anti-ferroptotic factor, was transcriptionally suppressed in Ovx mouse femurs due to DNMT1/3a/3b elevations and subsequent GPX4 promoter hypermethylation; (2) GPX4 suppression was co-regulated by repressive KLF5 and transcriptional co-repressors NCoR and SnoN; (3) The DNMT inhibitor SGI-1027 effectively reduced the GPX4 suppression and mitigated the ferroptotic bone loss in Ovx mice; (4) The GPX4 suppression and ferroptosis occurred mainly in osteoblasts and osteoblast-specific GPX4 semi-knockout (*Gpx4*^-/+^) incurred spontaneous and exacerbated ferroptotic OP alterations in Ovx mice, and finally (5) GPX4 pharmacological inactivation or semi-knockout in osteoblasts significantly diminished the protective effect of SGI-1027 against DNMT-mediated ferroptotic bone loss. Thus, our data indicate that the DNMT1/3a/3b aberration-incurred GPX4 suppression and the consequential osteoblastic ferroptosis causally impact the development of OP, which can be effectively targeted by DNA demethylating agents for therapeutic benefits.

The identification of DNMT1/3a/3b as the critical initiators of GPX4 suppression and ferroptosis critical for OP is an important discovery of this study. It is well known that both ferroptosis and OP are affected by epigenetic DNA methylation aberrations,^29,42^ however the mechanistic link between these two events has been missing. Recent studies suggest that GPX4 suppression due to aberrant DNA methylation might be a decisive event in ferroptosis under certain ferroptotic conditions.^24,25^ The actual methylation status of a particular gene promoter is regulated by a number of factors, including methyl donors, DNA insulators, DNA methylation “writer” DNMTs, “reader” methyl-CpG-binding domain (MBD) proteins and DNA demethylating TET enzymes.^43–45^ The initial “drivers” of the DNA methylation alterations in OP are unclear. Previous studies from our and other labs have reported that aberrant DNMT elevations causally affected OP.^46–49^ In this study, we detected that the GPX4 suppression occurred substantially at the transcriptional level that correlated with increased expression of all three bioactive DNMT isoforms and GPX4 promoter hypermethylation in the distal femurs of both OP patients and Ovx mice. We further demonstrated that individual knockdown of DNMT1/3a/3b significantly reduced the GPX4 suppression and lipid peroxidation in osteoblasts, and the pan-DNMT inhibitor SGI-1027 effectively corrected the GPX4 promoter hypermethylation, GPX4 suppression and the ferroptotic OP alterations in Ovx mice, which were abrogated when GPX4 was genetically-deficient or pharmacologically-inactivated (Fig. 8). These results strong evidence that DNMT1/3a/3b aberration-driven GPX4 suppression and ferroptosis contribute substantially to OP pathologies.

Osteoferroptosis has been reported in OP animals of various etiologies, including estrogen-deficient OP modeled by Ovx mice, glucocorticoid-induced OP and diabetic OP, although with conflicting resultson the expression of the central ferroptosis regulators NRF2 (nuclear factor erythroid 2-related factor 2), GPX4 and HO1 (heme oxygenase-1),^50–52^ which require further clarification. Ferroptosis can affect almost all bone resident cells including bone marrow mesenchymal stem cells,^53^ immune cells,^54^ osteocytes^50,51^ osteoblasts^52,55^ and osteoclasts.^56,57^ In fact, ferroptosis of osteocytes and osteoblasts is known to causally influence OP,^50–52^ which is conceivable since osteocytes are differentiated from osteoblasts^55^ and due to the inheritable nature, epigenetic ferroptosis of osteoblasts is likely to influence the phenotypes of osteocytes. In this study, we showed that GPX4 suppression and ferroptotic alterations mainly occurred in osteoblasts, as demonstrated by immunofluorescent double-staining of femoral sections and various assays in primarily-cultured osteoblasts. These osteoblasts also displayed typical ferroptotic alterations such as GPX4 suppression, abnormal lipid peroxidation and mitochondrial morphological changes that were absent from osteoclasts. This is further confirmed by osteoblast-specific *Gpx4* deficient mice that developed spontaneous and worsened ferroptotic OP pathologies in Ovx mice. Our results are consistent with the consensus that reduced osteoblast number or activity interrupts the balance between osteoblastogenesis and osteoclastogenesis and leads to OP.

The revelation of KLF5 as a transcriptional co-regulator critically involved in GPX4 suppression has provided insightful information on the mechanisms of DNA methylation-mediated gene silencing. Gene transcriptional inhibition induced by DNA methylation is mediated by a transcriptional repressive complex containing multiple transcriptional repressors and co-regulators that provide gene specificity.^28^ KLF5 is well documented for its regulation of multiple cellular processes, including the cell cycle and proliferation, apoptosis and autophagy, migration and invasion, and stemness and differentiation via either positively or negatively regulating the transcription of target genes.^36^ We found that the GPX4 promoter contains a strong KLF5 motif and KLF5, together with the transcriptional co-repressor NCoR and SnoN, inducibly bound to the hypermethylated GPX4 promoter in OP femurs that is sensitive to DNMT inhibition (Fig. 3). We further showed that KLF5 inhibition by ML264 partially relieved the GPX4 suppression in osteoblasts. These data support that KLF5, NCoR and SnoN may be the core components of the transcriptional repressive complex that mediates the DNA methylation-incurred GPX4 transcriptional suppression in OP.

It is noteworthy that our study has revealed a novel epigenetic feature of osteoblast ferroptosis in OP, but also raised additional questions. For example, GPX4 suppression is affected by many known and unknown ferroptotic stimulations, it is unclear which upstream network actually induces DNMT1/3a/3b aberrations and whether DNMT aberration-induced ferroptosis is unique to Ovx mice or a general character of all etiologies of OP. Furthermore, it is also unclear how epigenetic DNMT alterations differentially affect osteoblasts versus osteoclasts under OP conditions. These questions warrant further investigation.

In conclusion, the results of our study demonstrate that aberrant DNMT1/3a/3b elevations and subsequent GPX4 suppression play a decisive role in osteoblast ferroptosis that contributes significantly to OP. Since epigenetic modifications are reversible and DNA demethylating drugs such as 5-azacytidine and 5-aza-2-deoxycytidine (decitabine) are approved for the clinical treatment of myelodysplastic syndromes and chronic myelomonocytic leukemia,^58^ our results also suggest that targeting epigenetic GPX4 suppression and ferroptosis through DNMT intervention could be a feasible and effective strategy for treating patients with OP and related bone diseases.

## Materials and methods

### Animal and treatment

The use of animals and the experimental protocols were approved by the Animal Care Committee of Nanjing University in accordance with the Institutional Animal Care and Use guidelines (2020AE01113). C57BL/6 J mice were from Gempharmatech Co., Ltd., Nanjing, China. We intended to generate osteoblastic *Gpx4* knockout mice by crossing *Gpx4*-flox mice (*Gpx4*^fl/fl^, Strain NO. T050827) and *Col1a1*-Cre mice (Strain NO. T004734, both were of C57BL/6 J background, GemPharmatech, Nanjing, China), but only obtained *Gpx4* haplo-deficient *Gpx4*^Ob+/−^ progenies. PCR genotyping was performed on mouse toe DNAs with primers F1: GTACTGCAACAGCTCCGAGTTC; R1: ACTTATCCAGGCAGACCATGTG; R2: AACTCCAATTCCCAGGACTCAC as depicted in Fig. 7a. Mice were housed under specific pathogen-free and standard (25 ± 2) °C, (50 ± 5)% humidity and a 12 h/12 h light/dark cycle conditions.

A mouse OP model was established using a bilateral ovariectomy protocol.^33^ Briefly, the experimental mice were anesthetized with isoflurane and the bilateral ovaries were removed through a midline incision of the skin and flank incisions of the peritoneum. The skin incision was then closed with metallic clips. Sham operation was performed similarly without removing the ovaries. For intervention study, female C57BL/6 J mice around 10-12 weeks old were divided into four groups: (1) Sham surgery group; (2) SGI-1027 (2.5 mg/kg, HY-13962) or ferrostatin-1 (Fer-1, 5 mg/kg, HY-100579) from MCE, USA, dissolved in 2% DMSO, 30% PEG 300 and 2% Tween 80 administered by daily intraperitoneal injection; (3) Ovx group; (4) SGI-1027/Fer-1 intervention group. For the mouse assay to determine the role of GPX4 in the SGI-1027 intervention, the experiments started with comparison between control and RSL3-treated (100 mg/kg, HY-100218A, MCE, USA) C57BL/6 J mice, and then between *Gpx4*^fl/fl^ and *Gpx4*^Ob+/−^ mice. All mice underwent Sham, Ovx and SGI-1027 intervention as described above. The experiments lasted six weeks, then the mice were sacrificed by excessive isoflurane and the femurs were collected and stored at -80^o^C, or treated with paraformaldehyde for further analysis.

### Human samples

Human samples were collected from Northern Jiangsu People’s Hospital, The Teaching Hospital of Nanjing University Medical School. OP is defined as a T score of ≤ −2.5, and a T score of ≥ −1 is considered normal bone density according to the National Osteoporosis Foundation. Osteoporotic lumbar were obtained from 6 female patients with lumbar fracture who received percutaneous vertebroplasty (62–82 years old) with an average bone densitometry T score of −3.9 ( − 3.6, −4.8, −4.4, −4.5, −3.8 and −2.8). The control non-OP lumbar were from six age-matched patients receiving internal fixation treatment (58–79 years old) with an average T score of −0.5 ( − 0.3, −0.6, −0.2, −0.6, -0.3 and −0.9). T scores were determined by dual energy X-ray absorptiometry. Samples were stored at −80 °C before further protein, histology and MSP analyses. The study was approved by the ethics committee of Northern Jiangsu People’s Hospital (2023ky232), and written informed consent was received from all subjects. Patients or members of the public are not involved in the design, conduct, reporting, or dissemination plans of the research.

### Bone micro-CT analysis

Trabecular microstructure analysis was performed as described previously^59^ with freshly-removed mouse right femurs fixed in 4% paraformaldehyde for 24 h. The micro-CT scanner (Scanco Medical, Bruettisellen, Switzerland) was set at 55 kV, 145 μA and 15.6 μm voxel with 250 ms integration time. Femoral mid-diaphysis above the growth plate and distal metaphysis were selected as the region of interest (ROI). For each sample, a total of 100 slices were evaluated to generate the three dimensional (3D) trabecular images, and trabecular volume to total bone tissue volume ratio (BV/TV), trabecular number (Tb.N), trabecular thickness (Tb.Th) and trabecular separation (Tb.Sp) were calculated.

### Histological, immunohistochemical (IHC), Perl’s Prussian blue, TUNEL and immunofluorescent staining

Mouse femur sections were processed with hematoxylin and eosin (H&E), Perl’s Prussian blue or IHC staining essentially as described before.^60^ For IHC staining, the sectioned slides were incubated overnight at 4 °C with primary antibodies against GPX4 (A11243, Abclonal, China), osteocalcin (OCN, 23418-1-AP, Proteintech, China), and then with HRP-conjugated secondary antibodies. Afterward, slides were processed with a DAB horseradish peroxidase color development kit (PR30010, Proteintech, china) and counterstained with hematin, IHC Profiler plug-in in Image J was used to automatically score staining intensity of samples (High positive 3, Positive 2, Low Positive 1 and Negative 0). Perl’s Prussian blue staining (G1029, Servicebio, china) and TUNEL (TdT-mediated dUTP Nick-End Labeling, A111-01, Vazyme, China) followed the instructions in the kits. The percentages of TUNEL-positively-stained cells over total cells from 10 randomly-selected fields were counted in a double-blinded manner.

Immunofluorescent double-staining of murine femur sections was performed essentially as before.^61^ Femur sections were first incubated overnight with the primary antibody mouse anti-GPX4 (67763-1-Ig, Proteintech, China) plus rabbit anti-OCN or rabbit anti-CTSK (11239-1-AP, Proteintech, China), respectively. The next day, the sections were incubated with secondary antibodies CoraLite488-conjugated Goat Anti-Rabbit IgG (SA00013-2, Proteintech, China) and CoraLite594-conjugated goat anti-mouse IgG (SA00013-3, Proteintech, China) followed by nuclear DAPI (C1005, Beyotime, China) staining. A confocal laser microscope (Olympus, Tokyo, Japan) was used to capture image.

### RNA sequencing data analysis

We downloaded the transcriptome data of bone tissue from Sham and Ovx mice (https://ngdc.cncb.ac.cn/gsa/browse/CRA007214). Reads from mouse data were aligned to the mouse genome GRCm38/mm10 using hisat 2.1.0. SAM files were sorted and converted to BAM using samtools v1.4. Reads with QS < 20 were excluded. For each sample, unique map reads with a map quality score ≥ 20 were reserved for subsequent analyses. HT Seq Python package (version 0.9.1) was used to count the number of reads of a unique map for each gene. The DESeq2 R package was used to perform differential expression analysis. Differentially expressed genes (DEGs) were assessed by log_2_ FC (fold change) ≥1 and *P* value < 0.05.

### Transmission electron microscopy (TEM) examination

Fresh mouse femurs or osteoblasts after various treatments were placed in a fixative containing 2% PFA and 2.5% glutaraldehyde (G5882, Sigma-Aldrich, USA), rinsed sequentially according to a conventional TEM sample preparation protocol, and fixed again in 1% osmium tetroxide. After dehydration and embedding in Epon812 (45345, Sigma-Aldrich, USA), Ultrathin sections and cells were stained with lead citrate and uranyl acetate and observed under a JEOL-1200EX microscope (Japan) in Shandong Weiya Laboratory, China.

### Primary cell culture

Primary osteoblasts were extracted from 3-day-young mice as previously described.^62^ Briefly, calvarial tissues were dissected and digested with 0.1% collagenase type I (SCR103, Sigma, USA) for three rounds, and the final cell pellets were collected and cultured in fresh α-MEM medium (SH30265.01B, HyClone, USA) with 10% FBS (FSD500, ExCell, China) and 1% penicillin/ streptomycin(15140122, Gibco, USA). For osteoblast differentiation, the culture medium was changed to osteogenic medium (PD-003; Procell, Wuhan, China), which contain 50 μg/mL ascorbic acid, 5 mmol/L β-glycerophosphate, and 10 nmol/L dexamethasone.

For primary osteoclasts, bone marrow monocytes (BMMs) were isolated from 3-week-old mice by flushing the bone marrow of long bones and cultured in complete α-MEM medium with 10% FBS and M-CSF (macrophage colony-stimulating factor, 30 ng/mL, CB34, Novoprotein, China) as before.^63^ Three days later, RANKL (Receptor activator for nuclear factor-κB ligand, 50 ng/mL, CR06, Novoprotein, China) was added to induce osteoclast differentiation.

### ALP or TRAP activity staining

For osteoblast ALP activity assay, cells were treated with FAC for 48 h, washed with phosphate-buffered saline (PBS) and then fixed with 4% formaldehyde for 15 min. After rinsing with PBS, cells were stained for ALP activity using a BCIP/NBT ALP color development kit (Beyotime, Nanjing, China). For osteoclast TRAP activity assay, the differentiated primary osteoclasts treated with FAC for 48 h or mouse femur sections fixed with 4% paraformaldehyde were stained for TRAP activities using a commercial kit (Sigma, 387A-1 KT, USA) according to the manufacturer’s instructions. For quantification, the positively stained cell areas over whole fields of 5 pre-set fixed locations were quantified using Image J software. For mouse femur section staining, the sections were counterstained with methyl green and the average number of TRAP-positively stained cells in 10 randomly selected fields was calculated in a double-blind manner.

### C11-BODIPY staining

A fluorescent radioprobe C11-BODIPY(581/591, D3861, Thermo Fisher, USA) was used to assess lipid peroxidation in cultured osteoblasts. Briefly, primary osteoblasts inoculated in 6-well plates under various treatments were treated with C11-BODIPY dye (10 μmol/L dissolved in DMSO) for 1 hour at 37^o^C in the dark. After repeated washes, cells were observed under a confocal fluorescence microscope with excitation/emission wavelengths of 488/510 nm for oxidized BODIPY (green) and 581/591 nm for non-oxidized BODIPY (red). Average fluorescence intensities were calculated based on six randomly-selected view fields, adjusted for cell numbers using Image J software.

### Western blotting

Western blot assays of mouse hind limb bones or cell homogenates were performed essentially as previously.^64^ The primary antibodies used were: NFATc1(AF06823) and DNMT3b (AF300068) from AiFangbiological, China; Col1 Collagen I (Col1, GB114197) and OPN (Osteopontin, GB112328) from Servicebio, China; GPX4 (A1933) and β-actin (AC026) from ABclonal, China; 4-HNE (4 Hydroxynonenal, ab46545, Abcam, Cambridge, UK); MDA (malondialdehyde, abx445120, Abbexa, Cambridge, UK); CTSK (DF6614), KLF5 (AF7542), KFL2 (DF13602), NCoR (AF0270) SMRT (DF8896), SnoN (DF3088) from Affinity Biosciences, China. Horseradish peroxidase (HRP)-conjugated secondary antibodies were from Proteintech, China. Western blots were visualized with fully automated chemiluminescence image analysis system (5200, Tanon, China) and protein levels were analyzed using Image J software.

### Methylated specific PCR (MSP) and bisulfite-sequencing PCR (BSP)

Prediction of CpG islands in the *Gpx4/GPX4* promoters and primer design for methylation-specific PCR (MSP) and bisulfite-sequencing PCR (BSP) were performed using online MethPrimer software (www.urogene.org/methprimer). The DNeasy Blood & Tissue Kit (69504, QIAGEN, Germany) was used to isolate total DNA. A DNA bisulfite conversion kit (DP215, TIANGEN Biotech, China) was used to convert unmethylated cytosine to uracil according to the manufacturer’s instructions. MSP and BSP were carried out according to previously-established protocols.^65^

The methylation levels of the mouse *Gpx4* promoter were assayed by MSP with the methylated forward primer 5ʹ-TTTTTTAAGGGGATGATTTTGATAC (−247/−223) and the reverse primer 5ʹ- ATACCCAATAATAAAAACGCGAA (−78/−100); unmethylated forward primer 5ʹ-TTTTAAGGGGATGATTTTGATATGT (−245/−221) and reverse primer 5ʹ-CATACC CAATAATAAAAACACAAA (−77/−100); and input DNA control forward primer 5ʹ-CTCTTTAAGGGGATGACTTTGACAC and reverse primer 5ʹ-ATGCC CAGTGATAGGGACGCGGG. The methylation levels of the human *GPX4* promoter were examined by MSP with the methylated forward primer 5’-AGTATTTTT AGGTTGTTTGGTTTGC (7/33) and the reverse primer 5’-CGAACGTACGAACTTA TTATTAACGA (152/179); the unmethylated forward primer 5ʹ-GTATTTTTAGGTT GTTTGGTTTGTG (8/34) and the unmethylated reverse primer 5ʹ- CAAACATACA AACTTATTATTAACAAC (152/180); and input DNA control forward primer 5ʹ-TAGACACAAGCGA GCATGCGCAGTC and reverse primer 5ʹ- CCAGAGCGCTCA TTGGTCAGACG. The PCR products were analyzed on 2% agarose gels and visualized under ultraviolet light, and densitometric analysis was performed using ImageJ software. The PCR part of BSP was performed with forward primer 5ʹ- GTTTTTTAAGGGGATGATTTTGATA (−248/−224) and reverse primer 5ʹ- CCCTACAA CCAATAAA AAACTAAATA (5/−22). PCR products were separated by electrophoresis and the target PCR fragments were purified and cloned into pGEM T Easy Vector (A1360; Promega). Five colonies from each mouse/PCR reaction were randomly selected for sequencing and the percentages of methylated cytosines over the total number of cytosines within the cloned fragment were calculated.

### Reverse transcription-polymerase chain reaction (RT-PCR)

Total RNA from mouse and human bone tissues was extracted essentially as before.^66^ After cDNA synthesis, PCR was performed with following mouse *Gpx4* primers *Gpx4*F: CCCATTCCTGAACCTTTCAA and *Gpx4*R: GCACACGAAACCCCTGTACT; *Actb*F: GATCATTGCTCCTCCTGAGC and *Actb*R: TGCACCGCAAGTGCTTCTA as internal control and human *GPX4* primers *Gpx4*F: GAAGCAGGAGCCAGGGAGTA and *Gpx4*R: ATGGCATTTCCCAGGATGCC; *ACTB* primers *ACTB*F: GCCTTCCTTCCTGGGCAT and *ACTB*R: CTTCATTGTGCTGGGTGCC. PCR products were separated on a 1.5% agarose gel and analyzed using Image J software.

### Chromatin immunoprecipitation (ChIP)

ChIP assay was performed on mouse femur tissue as before.^67^ Immunoprecipitation was performed with ChIP quality antibodies to KLF5, NCoR, SMRT and SnoN. The starting (input) and immunoprecipitated DNAs were analyzed by PCR and quantitative real-time PCR (qRT-PCR) using forward primer 5ʹ-GGGGATGACTTTGACACGC and reverse primer 5ʹ-GCCTGAATGAAGGGACGG, which covered the −239 to −14 locus of the *Gpx4* promoter containing a putative KLF5 binding motif (−43/gccccgccca). Regular PCR products were separated on 1.5% agarose gels and analysis of PCR product densitometry were performed using Image J Software.

### Luciferase assay

Primary osteoblasts were transiently transfected (FuGENE® HD Transfection Reagent, Promega, USA) with the *Gpx4* promoter reporter plasmid *Gpx4*p-luc (containing 2 000 bp of the *Gpx4* proximal promoter, customer-made by Genechem, China) and a Renilla luciferase reporter (Genechem, China) as internal control. After the transfected cells received various treatments, the luciferase activities from cell lysates were assayed using a dual luciferase reporter assay kit (Promega, USA). The luciferase activities of *Gpx4*p-luc were normalized to Renilla luciferase levels and expressed as relative fold changes.

### Statistical analysis

Data normal distribution and assumption of homogeneity of variances were assessed by Shapiro-Wilk test and Levene’s test, respectively. Data quantification and graphic plotting were performed using GraphpadPrism. Effect size η^2^ (large effect size, η^2^ ≥ 0.137 9; medium effect size, 0.058 8 ≤ η^2^ < 0.137 9; small effect size, 0.009 9 ≤ η^2^ < 0.058 8) were calculated using SPSS V22.0 software. Data were expressed as mean ± SEM for animal studies or ± SD for cell assays. Box-and-whisker plots were defined as follows: midline represents the median, box is the 25th-75th percentile and whiskers are the minimum and maximum. Group differences were analyzed by Student’s *t* test, two-way ANOVA, or two-way ANOVA followed by Tukey’s post hoc test. The thresholds of *P* < 0.05 were set as statistically significant.

## Supplementary information

Supplemental Figure

## Data Availability

Raw data are available from the corresponding authors on reasonable request.
