# Supplementary material for: DNMT aberration-incurred GPX4 suppression prompts osteoblast ferroptosis and osteoporosis
Source: Bone Res. 2024 Dec 2;12:68. doi: 10.1038/s41413-024-00365-1 (PMC11609303; doi:10.1038/s41413-024-00365-1)
Supplement: Supplementary file 1 — Supplemental Figure [file 41413_2024_365_MOESM1_ESM.pdf]

Figure S1

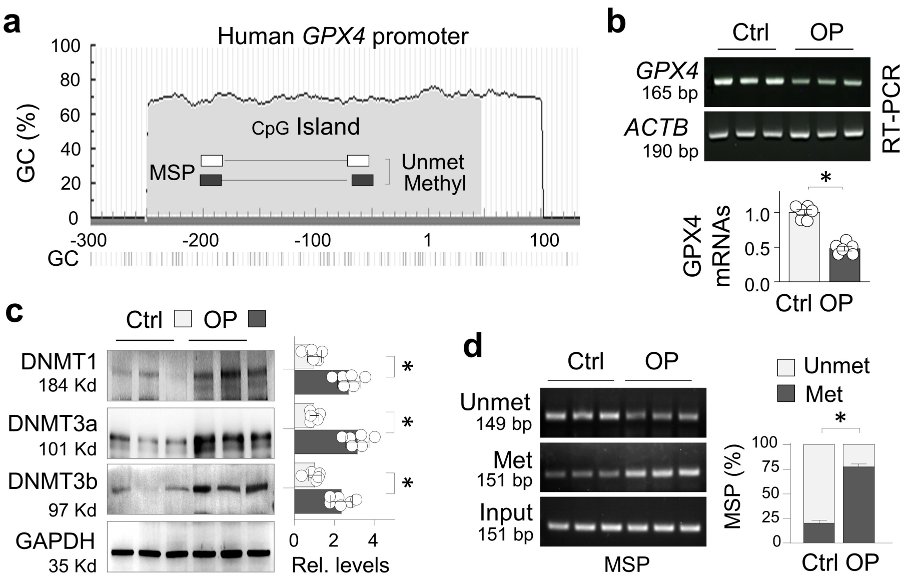

Supplemental figure S1. GPX4 is transcriptionally suppressed in femurs of OP patients, with concurrent DNMT elevation and GPX4 promoter hypermethylation. **(a)** A SchEMatic diagram of human *GPX4* promoter. The positions of CpG island (grey area), CpG sites (GC), and MSP primers (boxes) were depicted relative to the transcription starting site. **(b)** RT-PCR and agarose gel analysis of *GPX4* mRNAs from control (Ctrl) and OP bone tissues. Beta-actin gene (*ACTB*) served as control. Three random samples from each group (n=6) were shown. Quantification bellow the gel figures was presented as mean ± SEM. **(c)** Western blotting of the bone homogenates from Ctrl and OP patients for DNMT1, DNMT2 and DNMT3 with GAPDH serving as control. Quantification on the right side was presented as relative mean± SEM. **(d)** Representative agarose gel analyses of MSP products (methylated, unmethylated and input PCR) from Ctrl and OP patients. Quantification on the right side was presented as mean ratio ± SEM of methylated/unmethylated over total PCR products after adjusted with input control. *P <0.05, Student's t test.
